# 

**Published:** 1895-10

**Authors:** 


					﻿Vol. XV.
OCTOBER, 1895.
Ml 16. ,
THE
HOMEOPATHIC pHY^lClAp,
A Monthly Journal of Homoeopathic Materia Medica
and Clinical Medicine.
“ He is a freeman whom truth make* free, and aU are slaves beside."
"Seek the Truth: come whence it may, cost what it will."
EDITED AND rUBLISHKD BY
WALTER M. JAMES, M. D.
PHILADELPHIA :
USTo. 1231 LOCUST STIRZEZHT.
X, p 29* xr p :pi :
ALFRED HEATH & CO., Sole Agents fob Cheat Britain,
' 114 Ebury Street, Eaton Square.
A®* Yearly Subscription, S2.SO, invariably in advance. Single numbers, tS etc.
Entered at tbe Philadelphia Post-OIDce as Second-Class Mail Matter,
				

